# Moods as ups and downs of the motivation pendulum: revisiting reinforcement sensitivity theory (RST) in bipolar disorder

**DOI:** 10.3389/fnbeh.2014.00378

**Published:** 2014-11-03

**Authors:** Tal Gonen, Haggai Sharon, Godfrey Pearlson, Talma Hendler

**Affiliations:** ^1^Functional Brain Center, Wohl Institute of Advanced Imaging, Tel Aviv Medical CenterTel Aviv, Israel; ^2^School of Psychological Sciences, Tel Aviv UniversityTel Aviv, Israel; ^3^Sackler Faculty of Medicine, Tel Aviv UniversityTel Aviv, Israel; ^4^Psychiatry Department, Yale School of MedicineBaltimore, MD, USA; ^5^Olin Neuropsychiatry Research Center, Hartford HospitalHartford, CT, USA; ^6^Psychiatry Department, Johns Hopkins UniversityHartford, CT, USA; ^7^Sagol School of Neuroscience, Tel Aviv UniversityTel Aviv, Israel

**Keywords:** functional neuroimaging, motivation and affectives process, bipolar disorder, depression, system neuroscience

## Abstract

Motivation is a key neurobehavioral concept underlying adaptive responses to environmental incentives and threats. As such, dysregulation of motivational processes may be critical in the formation of abnormal behavioral patterns/tendencies. According to the long standing model of the Reinforcement Sensitivity Theory (RST), motivation behaviors are driven by three neurobehavioral systems mediating the sensitivity to punishment, reward or goal-conflict. Corresponding to current neurobehavioral theories in psychiatry, this theory links abnormal motivational drives to abnormal behavior; viewing depression and mania as two abnormal extremes of reward driven processes leading to either under or over approach tendencies, respectively. We revisit the RST framework in the context of bipolar disorder (BD) and challenge this concept by suggesting that dysregulated interactions of both punishment and reward related processes better account for the psychological and neural abnormalities observed in BD. We further present an integrative model positing that the three parallel motivation systems currently proposed by the RST model, can be viewed as subsystems in a large-scale neurobehavioral network of motivational decision making.

The term motivation as used in neuroscience refers to the processes which modulate the organism’s responses to environmental reinforcing cues, according to their perceived value (i.e., reward/punishment) (Smillie, 2008). As it is a major determinant of adaptive goal-directed behavior, it may be useful to look at human psychopathological conditions in terms of aberrant neuro-behavioral functioning of motivational processes. Accordingly, in this perspective paper we revisit a long-standing framework stemmed from rodent research, the Reinforcement Sensitivity Theory (RST; Gray, 1982), which defines sensitivity to reward and punishment as the main underlying forces of goal-directed behavior. Specifically, we inspect the neural organization proposed by the RST with respect to its possible involvement in a specific mood dysregulation condition- Bipolar Disorder (BD). We briefly review previous attempts to formulate a conceptual model linking motivation-related abnormalities and BD, and suggest an alternative perspective regarding the compound interactions between the different neural systems underlying motivational processes.

## Motivation systems and their relevance to affective disorders

**Figure 1:**
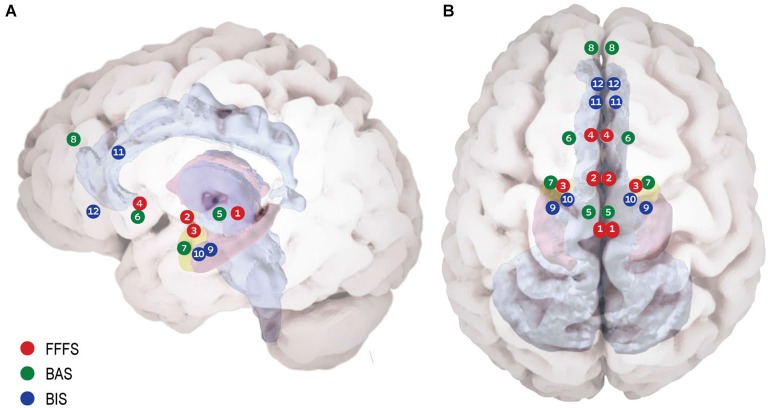
**Proposed neuroanatomy of the RST motivational systems. (A)** Lateral view. **(B)** Superior view. According to RST three bio-behavioral systems participate in reinforcement modulation of goal directed behavior: **(1) The Fight Flight Freeze System (FFFS)** is activated by all punishment stimuli (shown in red). The emotional consequence of its activation is fear, and the motivational consequence is defensive approach (i.e., fight) or defensive avoidance (i.e., flight/freeze). Anatomically, this system includes the Peri-Aqueductal Gray (1), Medial Hypothalamus (2), central Amygdala (3) and subgenual Anterior Cingulate Cortex (4). **(2) The Behavioral Activation System (BAS)** believed to underlie reward (and non-punishment) sensitivity (shown in green). When detecting reward, the system enhances incentive motivation, which facilitates approach. The system relies on Ventral Tegmental Area dopamine phasic activity (5) to Nucleus Accumbens (NAc) (6) in response to reward, signaling its salience. Information regarding the classical stimulus-reinforcement conditioning, along with integrative stimulus-reward associations is projected to the NAc from the basolateral amygdala (7). The medial pre-frontal cortex carries integrative representation of complex reinforcement associations with both stimuli and responses, is suggested to control and modulate incentive motivation and approach behavior (8). **(3) Behavioral Inhibition System (BIS)** underlying goal-conflict situations (shown in blue). The BIS is activated by stimuli of mixed valence (i.e., both BAS and FFFS are already activated), causing conflict between differing goals. The system functions as a comparator between the current state, previous knowledge and expected consequences, for the sake of adaptive behavioral selection. BIS consists of two neural foci: the Septo-Hippocampal System (SHS) (9) is informed comprehensively regarding possible behavioral plans for the current situation and their consequences by the entorhinal cortex (10) and cingulate cortex (11). The SHS is further modulated by information from the FFFS’s central amygdala, signaling the valance and importance of the stimuli. The ventro-medial pre-frontal cortex (12) is considered as a behavioral control modulator.

The basic concept regarding the consequential effects of stimuli on behavior, depending on their incentive values for the organism (reward or punishment) originated with the seminal work of Pavlov (Pavlov, 1927). Essentially, animals tend to approach rewarding and avoid punishing cues, creating the idea of sensitivity to reward and punishment as the main underlying forces of goal-oriented behavior (Corr and Perkins, 2006). Following this view the RST (Gray, 1982) assigned theses sensitivities to three specific neural systems mediating different motivational processes: (1) The “Fight, Flight, Freeze System” (FFFS), sensitive to punishment stimuli and facilitates behavioral responses via activation of the periaquaductal Gray (PAG), medial hypothalamus, central amygdala, and subgenual anterior cingulate cortex (sgACC); (2) The “Behavioral Activation System” (BAS), sensitive to reward stimuli and facilitates behavioral responses via the ventral tegmental area (VTA), nucleus accumbens (NAcc) and dorsomedial prefrontal cortex (dmPFC); and (3) The “Behavioral Inhibition System” (BIS), sensitive to goal-conflict situations (i.e., stimuli of mixed value) and thus triggered when the other two systems are simultaneously activated, relying mainly on the septo-hippocampal system (SHS) and the ventromedial prefrontal cortex (vmPFC) recursive circuit, along with the Anterior Cingulate Cortex (ACC) and entorhinal cortex (Gray and McNaughton, 2000). Figure 1 depicts the detailed neuroanatomy of these systems. Research on the neural dynamics and interactions of these proposed motivation systems has been mainly carried out in animals (rodents), with translation of the experimental paradigms to the complexity of human behavior somewhat lacking (Avila et al., 2008; Barrós-Loscertales et al., 2010; Costumero et al., 2013). With this in mind, in a recent fMRI study we used an interactive computer game and Dynamic Causal Model analysis to demonstrate the effective connectivity and stimulus specificity in similar neural networks in humans as described in the RST model (Gonen et al., 2012). This finding has encouraged us to revisit RST in light of current evidence on the relevance of motivation systems to psychopathology.

The relevance of motivation systems to abnormal mood conditions is supported by recent studies focusing mainly on reward processing. This has reflected developments in the investigation of the neural underpinnings of reward processes in terms of their neurochemistry (Berridge and Kringelbach, 2013) and mechanistic circuitry (Haber and Knutson, 2009; Miller et al., 2013). Furthermore, various aspects of motivation induced behaviors, such as reward learning (Pizzagalli et al., 2008; Dayan and Berridge, 2014), reward prediction (Dowd and Barch, 2012) or aversion processing (Hayes and Northoff, 2011) were proposed as related to psychopathological conditions (Phillips and Swartz, 2014). For example, altered effort-based motivational decision making has been demonstrated in Major Depressive Disorder (MDD) patients, who were not only willing to devote less effort for rewards than healthy controls, but were also less efficient in using information regarding the magnitude and probability of the rewards to guide effort based motivational decision making (Treadway et al., 2012). Such effort based decision making has been shown to rely on striatal dopamine transmission (Salamone and Correa, 2012) and its interaction with Ach muscarinic function, mostly in the NAcc core (Nunes et al., 2013). These findings have led to the idea that poor DA-ACh regulation within the NAcc may underlie common depressive symptoms such as anhedonia, fatigue or psychomotor slowness (Treadway and Zald, 2011). In the framework of the RST, these recent mechanistic evidence may provide new neurobiological support for involvement of the BAS in pathological affective states, since NAcc is a core region of this system. The contribution of abnormal motivational processes to human psychopathology in terms of the RST model has been formulated by two central models thus far. The first and most prominent is the “Neuropsychology Theory of Anxiety” (Gray and McNaughton, 2000), and the second is the “BAS Dysregulation Theory” (Depue and Iacono, 1989; Johnson et al., 2012). The first model posits that hyperactive nodes of FFFS, as well as BIS underlie different anxiety disorders (e.g., generalized anxiety, phobias etc.). A thorough discussion of the relation of RST to anxiety disorders is beyond the scope of this paper and can be found elsewhere (McNaughton and Corr, 2008). In the following section we will re-visit the BAS dysregulation theory and its limitations in thoroughly elucidating the underlying neural pathology in BD.

## Revisiting RST in the context of BD: dysregulated motivation processing

The “BAS Dysregulation Theory” suggests that it is BAS hyper- or hypo-activation that underlies BD manic or depressive states, respectively. Indeed, the model seems compatible with the nature of the symptoms often observed in BD patients. For example, decreased energy in depression or increased engagement in goal-directed activities in mania, support the consideration of motivational abnormalities when modeling these illnesses. Therefore, it seems quite straightforward that extreme over- and/or under- sensitivity to reward (manifested as over and under activation of the BAS) underlies mania or depression, respectively. Accordingly, numerous self-report studies using RST based questionnaires have shown higher BAS sensitivity in bipolar patients (Salavert et al., 2007) and also found it to be related to vulnerability to manic episodes (Meyer et al., 2007; Alloy et al., 2008). However, some have found relation of BIS sensitivity (measured by the self-report BIS/BAS scales) to depressive symptoms. For example, in a longitudinal study, within-patient fluctuations in depressive symptoms were correlated to changes in BIS levels (Meyer et al., 2001). Highly sensitive BIS was found related to proneness to- or concurrent depressive symptoms as well (Alloy et al., 2006). This inspires a reconsideration of the RST relevance to BD in a broader perspective of two processes, reward and punishment dysregulation.

In this context we suggest that dysregulation of the BIS system may result in insensitivity to costs and efforts, a symptomatic phenomenology of the manic state in BD. Recent evidence considering the inter-regional regulation of effort based behavioral choice via NAcc and ACC may support this notion. The ACC has been implicated in evaluating the costs and benefits and comparing between current and future costs /benefits of optional behavioral plans (Phillips and Vieta, 2007). Of note, the ACC has been shown to be involved in effort based decision processs only in cases where there were more than one potential reward (Schweimer et al., 2005), supporting its role in the RST’s BIS as evaluating optional behavioral plans under a goal conflict. However, while the RST postulated that motivational behavioral decisions are guided by the septo-hippocampal-vmPFC recursive signaling, with the ACC signaling information to the hippocampus; more recent models show in addition direct connections from the ACC to the NAC (Knutson and Gibbs, 2007; Phillips and Vieta, 2007). Lesions to the ACC or its afferent connections with NAcc have been shown to diminish the effort an animal is willing to invest in a reward (Schweimer et al., 2005; Phillips et al., 2007; Treadway and Zald, 2011).

**Figure 2:**
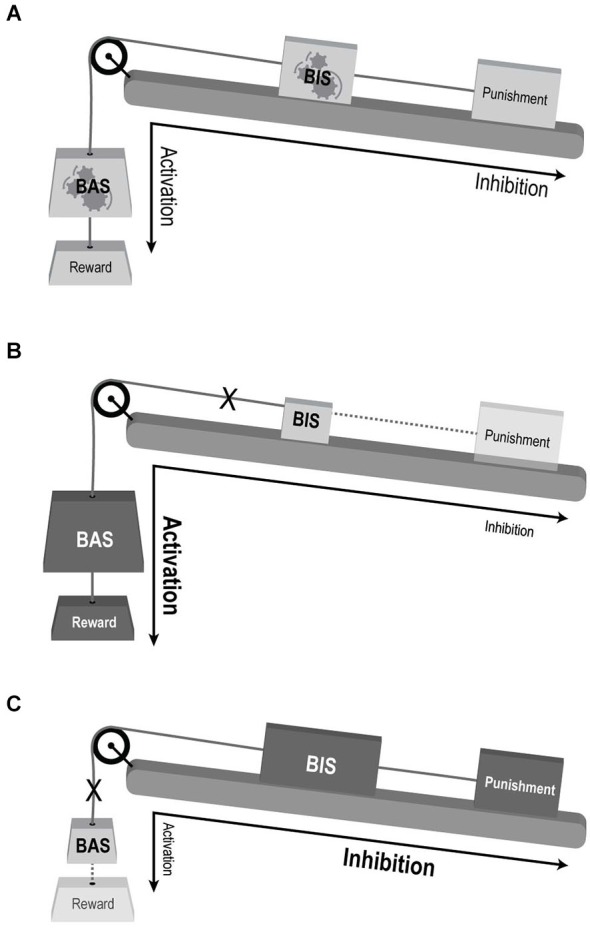
**Conceptual illustration of the suggested model for the involvement of motivational systems in mood disorders. (A)** Normal interaction. BAS mediating reward driven approach behavior and BIS mediating behavioral response to complex valence stimuli. Both sub-systems’ response and influence on behavioral output are balanced. The large-scale system is flexible and adaptive to changing motivational cues (denoted be gears within the “BAS” and “BIS” weights). **(B)** BAS over-activation enhancing reward sensitivity and approach behavior, and BIS under-activation reducing punishment sensitivity and avoidance—compatible with manic state. **(C)** BAS under-activation with reduced reward driven approach behavior, and BIS over-activation enhancing punishment sensitivity and avoidance—compatible with depression. Weight’s size and shading denotes activation levels: larger and darker weight indicate over activation, while smaller and lighter weights indicate under-activation. Dotted lines decipher weak impact of the reinforcer (punishment or reward) on the relevant system (BIS and BAS, respectively). X denoted disconnection of the sub-system from the large-scale motivational system.

Taken together, we feel that the BAS dysregulation model does not adequately capture the complex dysfunction of motivational processes in BD and that a conceptual revision is needed to better account for both phenomenological and neuroscientific observations in BD. Our premise echoes recent advances in neuroscientific research which repeatedly point to large-scale neural networks, rather than localized regions, as the underpinnings of cognitive and emotional processes (e.g., Bressler and Menon, 2010). We propose to regard the three motivational RST systems as functionally specialized subsystems of one larger system, interacting together in order to mediate motivational behavior. Thus, ineffective compound interactions between the three subsystems, rather than one subsystem’s abnormal activity, may underlie the different abnormal behaviors in mood disorders such as BD (see Figure 2). To establish this view, the following sections present a conceptual framework alongside supporting evidence from structural as well as functional neuroanatomical studies.

## Evidence for abnormal RST processes in BD

### Cognitive-behavioral findings

The clinical presentation of the manic state includes by definition, among other symptoms, an increase in goal-directed activity and excessive indulgence in pleasurable activities that have an increased potential for painful consequences; whereas depression is characterized by symptoms including anhedonia and fatigue or loss of energy. It is thus rather intuitive that extreme over- and/or under- reactivity of the BAS may underlie mania or depression, respectively, as suggested by the “BAS dysregulation theory” (Depue and Iacono, 1989; Johnson et al., 2012). However, there seems to be an important role for abnormal punishment sensitivity as well. Manic patients lack accurate perception of possible aversive consequences of their behaviors, especially the concept of punishment and its related results (Diekhof et al., 2008), leading to damaging interactions or consequences (Malhi et al., 2004a). Manic patients have also been shown to display positive cognitive bias (e.g., remembering more positive self-descriptive words than healthy controls (Pavlickova et al., 2013)). Nevertheless their behavior seems to be guided more by sensitivity to potential rewards than by avoiding dangers (Swann et al., 2004). We suggest that this complex positive cognitive-behavioral bias may reflect impaired generation, or resolution, of goal-conflict processes, which according to RST depends on normal activation of the BIS. Thus, in the search for the motivational processes underlying the manic state, BIS dysregulation should also be considered (as demonstrated in Figure 2B).

A parallel argument applies to the depressive state in BD patients in whom lack of incentive motivation is a prominent feature: depressed BD patients exhibit decreased reward seeking behavior and a reduced ability to experience reward, even in the absence of acute stressors, resulting in dominance of persistent dysphoric emotions and thoughts (Drevets, 2001), together corresponding to a hypoactive BAS. Other characteristics of the depressive state, such as the well-established negative cognitive bias, may hint at the involvement of a dysregulated FFFS/BIS (Leppänen, 2006; Haas and Canli, 2008; Zinbarg and Yoon, 2008; Figure 2C). Altogether it seems that one RST system’s abnormality is not sufficient to account for the complex behavioral abnormalities observed in BD. Rather, imbalanced interactions of both BAS and FFFS/BIS, resulting from over/under activation of one or both systems or from abnormal inter-system connectivity, can account for what seems to be both incentive and regulatory dysfunction. From this perspective, mania and depression can be viewed as two separate entities, rather than opposite ends of the same continuum along the BAS axis, a view that may also better account for their co-occurrence during mixed episodes (i.e., simultaneous episodes of mania and depression) (Cuellar et al., 2005).

### Brain imaging findings

Considerable evidence from morphometric MRI and post-mortem histological studies in BD patients demonstrate abnormalities in brain structures related to the three RST systems, thereby supporting the involvement of multiple dysregulated motivation systems and our integrative model (Diekhof et al., 2008; Savitz and Drevets, 2009). For example, the common finding of reduced gray matter volumes of the ACC (Phillips and Vieta, 2007; Strakowski et al., 2012) is compatible with disrupted function within a major BIS area. To note, these abnormalities have mostly been described in the sub-genual ACC, which is regarded as a central node of the FFFS (Pearlson, 1999; Fountoulakis, 2008; Sanches et al., 2008). In a similar manner, reduced gray matter volumes have been found in BD in several prefrontal cortices (PFC), spanning more than one motivation subsystem, including ventro-medial PFC for BIS (Doris et al., 2004; Lyoo et al., 2004), dorso-medial PFC for BAS (Locke and Braver, 2008) and Orbito Frontal Cortex (OFC; Haznedar et al., 2005; Lyoo et al., 2006), a region which some relate to BAS (Depue and Collins, 1999). Interestingly, Diffusion Tensor Imaging (DTI) analysis further demonstrated reduced integrity of white matter tracts in OFC (Beyer et al., 2005). Although the inter-segregation of the PFC is somewhat simplistic, given the multiple different processes these regions are involved in, these findings suggest possible deficits in PFC regulation and control functions affecting both BAS and BIS. To sum up the structural findings in the PFC—abnormalities were found in the sgACC related to the FFFS, the dmPFC and OFC related to BAS and in the vmPFC and ACC related to BIS.

Structural variations in amygdala and hippocampal volume are also common in BD (Strakowski et al., 2012; Phillips and Swartz, 2014), suggesting the involvement of BAS, FFFS and BIS. Yet, results in these regions vary with respect to the type of abnormality (e.g., Pearlson et al., 1997; Altshuler et al., 1998), possibly due to inter-study variations in medication regimes and comorbidities.

Several functional imaging studies using functional MRI (fMRI) indicate combined system involvement in BD (see summary in Table 1). Together these finding suggest two patterns of RST activations depending on BD states: hyperactivation of BAS along with hypoactivation in the FFFS/BIS in mania and vice versa depression.

**Table 1:** **Evidence from functional imaging studies**.

RST systems	State	Paradigm/Stimuli	Activation/Regions	Reference
FFFS	Manic	Emotional inhibition control	Hypo: sgACC	Elliott et al. (2004)
	Depressive	Affective generation (positive and negative)	Hyper: sgACC	Malhi et al. (2004b)
BAS	Manic	Rewarding, positive	Hyper: NAcc, caudate nucleus, OFC, dmPFC, amygdala	Yurgelun-Todd et al. (2000), Elliott et al. (2004), Lawrence et al. (2004), Altshuler et al. (2005), Chen et al. (2006), Wessa et al. (2007), Killgore et al. (2008), Bermpohl et al. (2010)
BIS	Manic	Emotional inhibition control	Hypo: vmPFC	Elliott et al. (2004)
	Depressive	Affective generation (positive and negative). Reward anticipation	Hyper: Parahippocampal gyrus	Malhi et al. (2004b), Deckersbach et al. (2008), Knutson et al. (2008)

This pattern has been convincingly demonstrated in the study by Blumberg et al. (2003) who investigated three BD patient groups: hypomanic, euthymic and depressed, while performing a Stroop task. Intriguingly, the hypomanic group showed decreased activity in the sgACC compared to the euthymic group, contrary to depressed patients who showed increased sgACC activity compared with the euthymic group, suggesting that FFFS, a punishment sensitive system, is under-activated in the manic state and over-activated in depression. In addition, the depressed group showed decreased dorsal and increased ventral activity in mPFC but the hypomanic group showed increased dorsal and decreased ventral activity in mPFC. These findings are compatible with decreased BAS and increased BIS activity in depression and vice versa in the manic state. This study thus points to deficits in prefrontal signaling in BD, possibly resulting from reduced FFFS-related punishment alerts, coupled with a diminished regulatory control of the BIS in manic state and vice versa in the depressed state. Further supporting this view is evidence from Positron Emission Tomography (PET) studies, showing increased sgACC, ACC and hippocampal metabolism in depressed BD patients during the resting state (Bauer et al., 2005; Mah et al., 2007).

Altogether, imaging findings strongly support our proposal for the involvement of several RST systems in BD, suggesting that rather than a deficit in BAS alone, the coupled interaction of BAS with BIS/FFFS is impaired (Figure 2). To note, this systems imbalance shows opposite directions for manic vs. depressed states, which may reflect a more general rule for explaining swings in mood states in other psychiatric conditions such as personality disorders.

## Discussion

We argue here for considering abnormal motivational processes in the pathophysiological study of mental illnesses, driven by neural system sensitive to reward, punishment or their regulation during goal-conflict. Based on a theoretical framework and converging empirical evidence, we suggest that although the BAS dysregulation theory has indeed provided a parsimonious and intuitive model for the involvement of motivational processes in BD, a conceptual revision is needed to further adapt these insights to the complexity of human psychopathology. Our new model maintains that since combined activity of the three motivation systems is responsible for the varied encounters with the external environment, BD is probably underpinned by compound motivational abnormalities: depression involves a hyperactive BIS and a hypoactive BAS, while mania results from a hyperactive BAS coupled with a hypoactive BIS (as illustrated in Figure 2). Notably, while it is possible that abnormal activity in BIS stems entirely from primary system abnormalities, deficits in FFFS activation may also be a contributing factor.

Thinking along this theoretical framework may provide useful insights into other psychiatric disorders as well. For example, similar abnormalities in motivation may also exist in unipolar depression MDD (Kasch et al., 2002; Kircanski et al., 2013), as was previously suggested (Eshel and Roiser, 2010).

Such a complex large-scale network model has recently become a common conceptual framework in neuroscientific research (Bressler and Menon, 2010) and especially with regard to emotional processing (Raz et al., 2012). Recent findings indeed point to abnormalities in large-scale network configuration in various psychopathologic states (e.g., Bassett and Bullmore, 2009), as we have recently discussed for the case of OCD (Hendler et al., 2014). By delineating the neurobiological underpinnings of basic psychological processes such as motivation and their dysfunction, this mechanistic approach may offer substantial progress in understanding the clinical presentation of mental illnesses and in treating them. This idea echoes the growing interest in the new approach termed Research Domain Criteria (RDoC; Sanislow et al., 2010) advanced by the NIMH, which aims to classify mental disorders based on dimensions of observable behavior and neurobiological measures. Indeed, within the framework of this approach motivational mechanisms were formulated into two RDoC domains: the “positive valence system” domain is related to approach motivation and reward processing, whereas the “negative valence system” domain is related to threat, loss and frustrative non-reward processing. The centrality of motivational processes in normal behavior attests to their probable involvement in pathological behavior as well. Inversely, looking at the complex abnormalities in motivation systems in human psychopathology may help determine the relations between these different constructs of motivation in normal behavior. Similar to studies in classic “lesion-neurology”, which advanced our understanding of normal brain function by exploring patients with focal brain lesions, the study of abnormalities of motivation as expressed across a range of pathological mental states may yield a deeper understanding of this primal yet complex neurobehavioral process.

To further investigate the core features of our model, such as dysfunction within and between motivational networks, future studies may explore the dynamics in functional-connectivity of the different systems’ key nodes using, for example, the network cohesion index (NCI; Raz et al., 2012), to reveal their ongoing interactions throughout performance in motivational tasks. Additionally, DTI studies may add the structural substrate to FC analyses in this effort. A causal role for different motivational circuits may also be demonstrated using interventional approaches. Recent developments in neurofeedback-based Brain Computer Interface (NF-BCI) using EEG and real-time fMRI methods aimed at modulating the activity in major nodes of different motivational systems may offer unique opportunities to track and validate the role of brain abnormalities in BD (Keedwell and Linden, 2013). Assessing the clinical efficacy of modulating a single vs. multiple motivation systems would further help to elucidate the underlying pathophysiology, and thus, may provide evidence regarding the suggested integrated model of motivational processes in the healthy brain as well.

## Conflict of interest statement

The authors declare that the research was conducted in the absence of any commercial or financial relationships that could be construed as a potential conflict of interest.
